# Gene Discovery of Characteristic Metabolic Pathways in the Tea Plant (*Camellia sinensis*) Using ‘Omics’-Based Network Approaches: A Future Perspective

**DOI:** 10.3389/fpls.2018.00480

**Published:** 2018-06-04

**Authors:** Shihua Zhang, Liang Zhang, Yuling Tai, Xuewen Wang, Chi-Tang Ho, Xiaochun Wan

**Affiliations:** ^1^State Key Laboratory of Tea Plant Biology and Utilization, Institute of Applied Mathematics, Anhui Agricultural University, Hefei, China; ^2^School of Life Sciences, Anhui Agricultural University, Hefei, China; ^3^Department of Genetics, University of Georgia, Athens, GA, United States; ^4^Department of Food Science, Rutgers University, New Brunswick, NJ, United States

**Keywords:** the tea plant, characteristic metabolic pathway, plant-specialized metabolite, transcriptomics, metabolomics, gene discovery, network approach

## Abstract

Characteristic secondary metabolites, including flavonoids, theanine and caffeine, in the tea plant (*Camellia sinensis*) are the primary sources of the rich flavors, fresh taste, and health benefits of tea. The decoding of genes involved in these characteristic components is still significantly lagging, which lays an obstacle for applied genetic improvement and metabolic engineering. With the popularity of high-throughout transcriptomics and metabolomics, ‘omics’-based network approaches, such as gene co-expression network and gene-to-metabolite network, have emerged as powerful tools for gene discovery of plant-specialized (secondary) metabolism. Thus, it is pivotal to summarize and introduce such system-based strategies in facilitating gene identification of characteristic metabolic pathways in the tea plant (or other plants). In this review, we describe recent advances in transcriptomics and metabolomics for transcript and metabolite profiling, and highlight ‘omics’-based network strategies using successful examples in model and non-model plants. Further, we summarize recent progress in ‘omics’ analysis for gene identification of characteristic metabolites in the tea plant. Limitations of the current strategies are discussed by comparison with ‘omics’-based network approaches. Finally, we demonstrate the potential of introducing such network strategies in the tea plant, with a prospects ending for a promising network discovery of characteristic metabolite genes in the tea plant.

## Introduction

The tea plant (*Camellia sinensis*) in the family *Theaceae* is an important commercial crop that is extensively cultivated in Asian, African, Latin American, and Oceanian countries (ITC, 2014). Leaves of this crop serve as the source of a popular non-alcoholic beverage known as “tea” due to its abundant production of many valuable secondary metabolites, such as polyphenols, alkaloids, theanine, vitamins, minerals, and volatile oils (Zhang et al., 2006, 2012; Chen et al., 2009; Sugimoto et al., 2009; Guo et al., 2011; Lu et al., 2011; Ghiringhelli et al., 2012; Weerawatanakorn et al., 2015). Among these small-molecular compounds, flavonoids, theanine, and caffeine represent the three major characteristic secondary metabolites that are main determinants of the rich flavors, fresh taste, and health benefits of tea (Yamamoto et al., 1997). For example, theanine and caffeine are the main taste compounds of green tea and contribute to the umami and bitterness, respectively (Narukawa et al., 2014). There have accumulated plenty of reports about the health benefits of EGCG, such as anti-oxidation, anti-inflammation, and anti-tumor (Tipoe et al., 2007). From the view of their biosynthesis, flavonoids originate from diverse branches of the phenylpropanoid pathway and include flavones, flavonols, isoflavones, flavanones, flavanols, anthocyanidins, and dihydroflavonols (Dixon and Pasinetti, 2010). Theanine biosynthesis starts from glutamine and pyruvate, and depends on the enzymatic processes of TS, GS, GLS, ALT, and ADC (Sasaoka et al., 1965). Caffeine is a purine alkaloid and its biosynthetic pathway comprises purine biosynthesis and purine modification steps (Li et al., 2015). In the tea plant, the disclosure of genes involved in the characteristic components biosynthesis is still lagging far behind the model *Arabidopsis thaliana* and even many non-model plants [e.g., sorghum (Blomstedt et al., 2015) and tomato (Verpoorte and Memelink, 2002)], which inevitably lays an obstacle to the potential applications in genetic improvement and metabolic engineering.

Recent advances in high-throughput phenotyping technologies, such as transcriptomics and metabolomics, have accumulated massive ‘omics’ datasets that quantify the expression/accumulation profile of transcripts/metabolites and facilitate the evaluation of interactions among these cellular components (transcriptional regulatory networks, metabolic pathways) and networks (Barabasi and Oltvai, 2004). In case of the associations of genes and plant-specialized (secondary) metabolites, two elements in cellular networks, namely nodes and edges, denote genes and/or metabolites and gene-to-gene and/or gene-to-metabolite interactions, respectively. In the past few decades, parallel and integrated analysis of the multi-‘omics’ datasets in a network fashion, such as gene co-expression network and gene-to-metabolite network, has become efficient ways to identify genes underling specialized metabolism in the model *Arabidopsis thaliana* and other non-model plants (Oksman-Caldentey et al., 2004; Hirai, 2009). Such gene discovery strategies are based on a simple assumption that genes involved in a specialized metabolic pathway are coordinately regulated under a shared regulatory system, using the ‘guilt-by-association’ principle (Oliver, 2000; Saito et al., 2008). With the large-scale ‘omics’ datasets generated in the tea plant, it is now a possible active area of gene discovery in characteristic secondary metabolism of this crop by borrowing the above-mentioned powerful ‘omics’-based network approaches.

With the above considerations, this review focuses on the agriculturally important crop, tea plant, in which the key genes of characteristic metabolites remains poorly understood. Firstly, we introduce recent advances in transcriptomics and metabolomics for transcript and metabolite profiling, and highlight different ‘omics’-based network strategies for the gene discovery in plant-specialized metabolism using successful examples that are applied in the model *Arabidopsis thaliana* and non-model plants (e.g., tomato, wheat). Further, we summarize recent progress in the ‘omics’ analysis for gene discovery of characteristic secondary metabolism in the tea plant, and limitations of the current strategies are discussed by comparison with ‘omics’-based network approaches. Finally, the potential of introducing ‘omics’-based network approaches in the tea plant are demonstrated, with a prospects ending for the promising network discovery of characteristic metabolite genes in the tea plant.

## Recent Advances in Transcriptomics and Metabolomics

### Deep mRNA Sequencing (RNA-Seq) for Transcript Profiling

Microarray and deep sequencing are useful technologies in transcript profiling (transcriptomics) due to their high-throughput and coverage (Malone and Oliver, 2011). In the tea plant, microarray has seldom been used because of the lack of biological resources (especially genomic sequences) that can aid in molecular probe design (Shi et al., 2011). In this review for the tea plant, phenotyping technology ‘transcriptomics,’ specifically RNA-seq (regardless of rRNA and non-coding RNA), can be applied in non-model species without reference genomes (Trapnell et al., 2012). There have accumulated many RNA-seq examples for the tea plant in the past 5 years. As a NGS technology, RNA-seq has become an efficient functional genomics tool in generating large-scale, low-cost mRNA expression data in model plants (*Arabidopsis thaliana* and rice) and non-model plants, such as crop (legumes, maize, and wheat), vegetables (cabbage and tomato), and trees (*Populus*) (Agarwal et al., 2014). Currently, several public repositories, such as Sequence Read Archive (SRA) at National Center for Biotechnology Information (Leinonen et al., 2010b) and European Nucleotide Archive (ENA) at European Molecular Biology Laboratory (Leinonen et al., 2010a), have made a vast volume of RNA-seq data available, serving as valuable resources for the data-driven knowledge discovery in plant secondary metabolism and many other fields.

A complete pipeline of RNA-seq consists of biological sample preparation, library construction, deep sequencing on a sequencing platform, and the downstream bioinformatics analysis (Wang et al., 2009). Routine analysis of RNA-seq data includes sequence alignment and/or *de novo* assembly, gene pathway and function annotation, gene expression qualification, and statistically identification of DEGs, which usually depends on whether a reference genome is sequenced for a species of interest (Conesa et al., 2016). Advanced analysis of RNA-seq data may be related to gene regulatory network reconstruction, gene module and motif analysis, and others (Iancu et al., 2012; Tierney et al., 2012). For experimental biologists without bioinformatics skills, there are several standalone softwares and online web-servers, such as RobiNA (Lohse et al., 2012) and TRUFA (Kornobis et al., 2015), for the easily-operated RNA-seq data analysis.

### Metabolomics Technology for Metabolite Profiling

Plants are a rich source of diverse specialized metabolites that have been used for a very long period as fragrances, flavors, colorants, insecticides, and pharmaceuticals (Facchini et al., 2012). A metabolome, known as a complete set of small-molecule metabolites in an organism, represents the resulting phenotype of cells deduced by the perturbation of gene expression, which are usually governed by external environmental changes (Saito and Matsuda, 2010). Therefore, metabolomics is of great importance in understanding cellular systems and decoding gene functions. A main concern in this field is metabolite profiling, i.e., targeted or non-targeted measurement of hundreds or potentially thousands of metabolites, such as amino acids, alkaloids, polyphenols, minerals, phenolics, and vitamins (Joyce and Palsson, 2006). To achieve this, a combination of sample extraction protocols, separation techniques such as GC and LC, and spectroscopic techniques such as MS and NMR spectroscopy are required for the quantitative and qualitative analysis of metabolites extracted from isolated plant cells or tissues (Kopka et al., 2004).

One of the disadvantages of current metabolomics is the limitation of public databases of metabolite accumulation in plants (include the tea plant) under different conditions; this is in striking contrast to transcriptomics for which many useful databases are readily available for the potential functional genomics research (Saito et al., 2008). To compensate this, several metabolome resources and tools have been recently developed. Among these, PMR (Bais et al., 2010), MPMR (Wurtele et al., 2012), and MeKO (Fukushima et al., 2014) are ‘metabolite profiling’-oriented databases that facilitate the sharing of comprehensive metabolome datasets in plants. In addition, organism-specific databases, such as MoTo DB (Moco et al., 2006) and SoyMetDB (Joshi et al., 2010), are now emerging in certain plant species of interest. Accompanying with these available metabolome resources, useful statistical and visualized tools, such as MeltDB (Neuweger et al., 2008), MBRole (Chagoyen and Pazos, 2011) and MetaMapp (Barupal et al., 2012), have also been developed to reinforce this promising field.

## A Survey of ‘Omics’-Based Network Strategies for the Identification of Specialized Metabolite Genes in Plants

### Introduction to Biological Network

**FIGURE 1:**
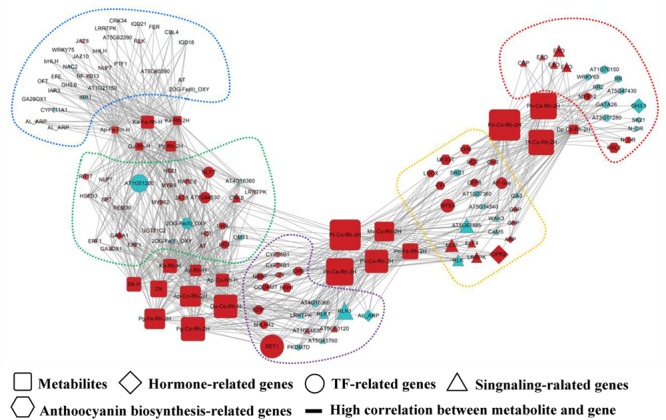
Schematic representation of a biological network. This diagram shows an example of a connection network between regulatory genes and anthocyanin-related metabolites (Cho et al., 2016). The network visualization is generated using Cytoscape software (Shannon et al., 2003), in which square, diamond, circle, triangle, and hexagon nodes denote metabolites, hormone-related genes, TF-related genes, signaling-related genes, and anthocyanin biosynthesis-related genes, respectively. An edge is placed between genes and anthocyanin-related metabolites indicating the gene-metabolite regulatory association. To be more informative, the node color and size are proportional the log_2_(fold change) of gene expression (metabolite accumulation) in comparison with controls, and different color of dashed frames indicate the modular structure of this network.

Biological networks are graph representations of molecular interactions in a biological cell system. A network can be defined as a set of nodes (or vertices), denoting metabolites, genes or gene products, and a set of directed or undirected edges (**Figure 1**), denoting the interactions between them (e.g., regulatory relationships, direct physical interactions, functional associations). The cell can be viewed as an overlay of at least three types of networks, which describes transcriptional regulations (directed), protein–protein interactions (undirected), and metabolic reactions (directed) (Alon, 2003). Similar to other naturally occurring networks such as those seen in computer science, power grids, social communication and the World Wide Web, biological networks have the characteristic topological organization, such as small-world and scale-free properties (Dorogovtsev and Mendes, 2013). In many fields of plant sciences including secondary metabolism focused here, it has been demonstrated that networking modeling using single- or multi-‘omics’ data has the possibility in capturing many of the essential characteristics of complicated biological cell systems (Hecker et al., 2009). More detailed information on biological networks, such as network reconstruction, visualization, and topological analysis, can be seen in several comprehensive review articles (Ravasz and Barabási, 2003; Barabasi and Oltvai, 2004).

### Gene Co-expression Network

After a glance of the basic concept of biological network, several types of ‘omics’-based network approaches for the identification of plant-specialized metabolite genes will be further introduced. The commonly used in this field is the application of transcriptome-based gene co-expression network (Higashi and Saito, 2013). In diverse types of biological networks, gene co-expression network can be attributed to a form of gene functional association network as it is inferred from the similarity of gene expression patterns across a wide array of experimental conditions. Publicly available datasets and in-house datasets can be used to compute the gene expression similarity, resulting in condition-independent and condition-dependent gene co-expression network, respectively [**Table 1** (Hirai, 2009)]. Different selection strategies of experimental conditions have been summarized by Usadel et al. (2009) for a comprehensive assessment. The measure commonly used for gene expression similarity is PCC because most secondary metabolism in plants proceeds along linear pathways and consists of irreversible chemical reactions governed by a single enzyme or a TF. Different measures used for gene expression similarity were summarized in **Table 2** and their advantages/disadvantaged have been discussed by Schaefer et al. (2017).

**Table 1:** Gene co-expression network classification based on experimental condition selection.

Network type	Transcriptome data source	Experimental condition	Gene co-expression correlation
Condition-dependent	In-house dataset	Specific condition of interest	Condition-biased
Condition-independent	Public dataset	A wide range of conditions	No bias

**Table 2:** Similarity measures used to calculate gene co-expression relationship.

Similarity measure	Statistic method	Computational efficiency	Gene co-expression relationship
Pearson correlation coefficient (PCC)	Parametric	Inexpensive	Linear
Spearman correlation coefficient (SCC)	Non-parametric	Moderate	Non-linear
Kendall correlation coefficient (KCC)	Non-parametric	Moderate	Non-linear
Mutual information (MI)	Non-parametric	Expensive	Linear and non-linear

**FIGURE 2:**
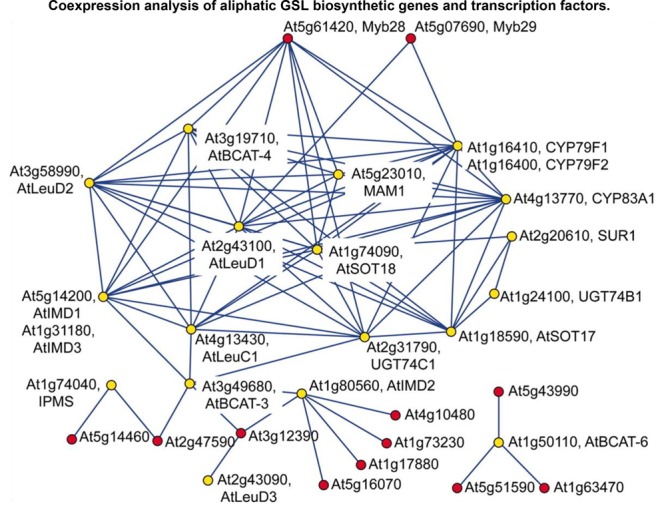
A gene co-expression network application in the gene discovery of plant-specialized metabolism. A gene co-expression module consisting of aliphatic GSL biosynthetic genes (yellow) and TF genes (red) is illustrated in this figure (Hirai et al., 2007), which was derived from the exhaustive analysis of co-expression between GSL genes and TF genes using a dataset of publicly condition-independent transcriptome profiles. This network visualization revealed that the known aliphatic GSL biosynthetic genes (as bait genes) were clustered in the same module together with two uncharacterized TF genes, *Myb28* and *Myb29*, suggesting that these two TF genes may be positive regulators of aliphatic GSL biosynthesis (experimentally validated in the following reverse-genetic and molecular experiments).

In systems biology, a logically conceivable assumption is that a set of genes involved in a particular biological process (more practically in a secondary metabolic pathway) are co-regulated and thus co-expressed under the control of a shared regulatory system. This is a typical example of the so-called ‘guilt-by-association’ principle. Based on this, gene co-expression network is widely utilized to identify genes in particular secondary metabolism in plants using guide-gene (i.e., bait gene) or non-targeted protocols (Aoki et al., 2007). In the model *Arabidopsis thaliana*, many enzyme genes and regulatory TF genes have been disclosed in specialized metabolic pathways, such as GSL [see **Figure 2** as a pioneering example (Hirai et al., 2007)], phenylpropanoid (Tokimatsu et al., 2005), cellulose (Brown et al., 2005), brassinosteroid (Lisso et al., 2005), hemicelluloses (Cocuron et al., 2007), flavonoid (Wei et al., 2006), and isoprenoid (Wille et al., 2004). Inspiringly, a large proportion of these candidate genes have been confirmed using reverse-genetic and biochemical experiments. Following these good examples, specialized metabolite genes have been continuously identified in non-model plants, such as *Cannabis sativa, Papaver somniferum, Solanum lycopersicum*, and *Catharanthus roseus* (Higashi and Saito, 2013). In public, there have appeared several co-expression databases and tools, such as ATTED-II (Obayashi et al., 2008), CSB.DB (Steinhauser et al., 2004), and GeneCAT (Mutwil et al., 2008) for model plant, primarily *Arabidopsis thaliana*, but also rice, poplar, barley, and others, which can be easily accessed for experimental biologists who have no any programming skills.

### Comparative Co-expression Analysis: From the View of Cross-Species Conversation

One possible caveat in gene discovery using gene co-expression relationships in single-species analysis is the risk of ‘false positives,’ i.e., co-expressed genes might be accidentally co-expressed rather than being functionally related. Generally, gene co-expression relationships in certain biological processes, such as cell cycle and protein synthesis and degradation, are conserved across diverse species (Stuart et al., 2003). Thus, two co-expressed genes from one species usually have their orthologs in another species that in turn are also co-expressed. According to this paradigm, the examination of conserved gene co-expression relationships across different species can be used to minimize the risk of ‘false positives’ via the knowledge transfer from model organisms to non-model plants (Movahedi et al., 2012). There are several tools, such as ATTED-II (Obayashi et al., 2011), CoP (Ogata et al., 2009) and StarNet (Jupiter et al., 2009), which allows between-species comparison of gene co-expression networks. In addition, the NetworkComparer pipeline deployed in PlaNet (Mutwil et al., 2011) can aid in multi-species comparison (Mutwil et al., 2011). This tool bins genes into gene families according to their Pfam annotation (Finn et al., 2016) and compares gene vicinity networks from the query genes for re-occurring Pfams. Using *AtPAL1* (responsible for the initial step for monolignol synthesis) as bait gene in NetworkComparer, Ruprecht and Persson (2012) inferred a consensus network of *AtPAL1*-orthologs in barley, *Medicago*, polar, rice, soybean, and wheat, from which gene families corresponding to the subsequent steps in lignin biosynthetic pathway, such as *4CL* (‘AMP_binding’), *C4H* (‘p450’), *HCT* (‘Transferase’), *CCoAOMT* (‘Methyltransf_3’), *CCR* (‘Epimerase’), and *CAD* (‘ADH_zinc_N’), were identified and experimentally confirmed.

### Gene Co-functional Network: From Single- to Multi-Network

As described above, gene co-expression network is a form of gene functional association network, which is only based on the similarity of gene expression patterns across a variety of experimental conditions. There exist other types of molecular relationships, such as physical interaction of proteins, subcellular co-localization, domain co-occurrence, co-citation in literature, mutant phenotypes, gene neighbors, genetic interactions, conserved motif sequences, and enzymatic reaction. These different levels of relationships are necessarily to be integrated in the original gene co-expression network to expand the network concept from single- to multi-network that can be called as gene co-functional network (Fukushima and Kusano, 2014). To increase the predictive power for gene identification, publicly available resources, such as AraNet (Lee et al., 2015), ATTED-II (Obayashi et al., 2011), and PlaNet (Mutwil et al., 2011), have integrated one or several of those valuable molecular relationships into the gene co-expression network, facilitating a more comprehensive gene co-functional network resource for users. It is noted that these efforts are limited to *Arabidopsis thaliana* because it is a data-rich model species (Lee et al., 2015). For other plants species, high coverage and confidence gene co-functional relationships can also be predicted via efficient computational pipelines. For instance, genome-wide gene co-functional networks have recently been integratively achieved in *Glycine max* (Kim E. et al., 2017), *Solanum lycopersicum* (Kim H. et al., 2017), and *Triticum aestivum* (Lee et al., 2017) by translating molecular interaction knowledge from data-rich model species (e.g., *Arabidopsis thaliana* and *Saccharomyces cerevisiae*) in them and using their in-species gene expression data.

### Gene-to-Metabolite Network: Co-occurrence Analysis of Genes and Metabolites

The metabolome in plant cell is the final product of a series of gene actions. Hence, metabolomics, when integrated with transcriptomics, provides a potential for the study of gene-to-metabolite networks that control specialized metabolism in plants, both at the catalytic and regulatory levels. Currently, this integrative analysis (especially the co-occurrence analysis in a network fashion) of gene expression and metabolite accumulation has emerged as an alternative strategy for the identification of novel gene functions involved in plant-specialized metabolism. Hirai et al. (2005) presented one of the very first articles successfully analyzing the gene/metabolite associations in a gene-to-metabolite network via the integration of time-series transcriptome and metabolome datasets. On the basis of known genes in GSL biosynthesis, the authors identified the following genes as candidates involved in GSL pathways: three putative sulfotransferase genes (At1g74100, At1g18590, and At1g74090), an *S*-glucosyltransferase gene (At1g24100), a putative Tyr aminotransferase gene (At5g36160), and two putative GST genes (At3g03190 and At1g78370). To date, some of these candidate genes have been experimentally characterized in concurrent studies using biochemical approaches (Grubb et al., 2004; Piotrowski et al., 2004).

## Recent Progress in ‘Omics’ Analysis for the Identification of Characteristic Metabolite Genes in the Tea Plant

### Transcriptome Analysis

We conducted the first RNA-seq based specialized metabolite gene analysis for the tea plant in the early 2011 through deep sequencing, *de novo* assembling and functional annotation of the transcriptome of a pooled sample of seven tissues including tender shoots, young leaves, mature leaves, stems, young roots, flower buds, and immature seeds (Shi et al., 2011). On this basis, many putative candidate genes involved in the three major secondary metabolic pathways (flavonoids, theanine, and caffeine) that tightly related to tea quality and taste were target-disclosed. Among these, several genes associated with theanine and flavonoid biosynthesis were experimentally validated using low throughout RT-PCR and qRT-PCR analysis. Generally, gene expression changes underlying specialized metabolite accumulation in plants at specific experimental conditions, developmental time points or different tissues is necessary to be measured for the disclosure of complicated regulatory mechanisms of specialized metabolites biosynthesis. In these cases, knowledge-gain aimed experimental designs (e.g., control/treat coupling and sample repetition) are preferentially considered to subject to the downstream data analysis namely DEG identification in routine RNA-seq analytical pipeline. Evidently, our effort is based on a multi-tissue composed mixture sample without the above biological concerns. With the popularity of RNA-seq analysis and increasing decrease of sequencing cost, many interesting hypotheses have been developed in the later 5 years by focusing specialized metabolism mechanisms of the tea plant in certain abiotic/biotic stress conditions [e.g., drought (Wang W. et al., 2016) and pathogen attack (Wang Y.N. et al., 2016)], different tissues [e.g., bud, stem, flower, and seed (Li et al., 2015)], and different developmental stages [e.g., leaf tissues at color-changing (Li et al., 2017) and form-shaping stages (Lin et al., 2017)].

### Comparative Transcriptome Analysis

Beyond the routine transcriptome analysis, comparative transcriptome analysis has become a valuable strategy in dissection of significant differences in genes and their expressions among different biological samples of a certain species, similar biological samples of different cultivars in a certain species, or even similar biological samples in different species. It is known that oil tea (*Camellia oleifera*) from the same genus *Camellia* lacks the three characteristic metabolites (flavonoids, theanine, and caffeine) that the tea plant (*Camellia sinensis*) predominately possesses. To uncover the genetic components underlying the biosynthesis of characteristic components in tea, we applied a cross-species transcriptome comparison by choosing bud and leave tissues from the two *Camellia* plants (Tai et al., 2015). Based on the RNA-seq analysis, we experimentally confirmed that several enzyme genes associated with flavonoid, theanine and caffeine pathways, such as PAL, CHI, DFR, and F3H, exhibited considerably different expressions in tea compared to oil tea using qRT-PCR analysis. Thus, it can be speculated that the differential expressions in certain genes behave as the contributing genetic basis for the divergence of metabolite contents in the two plants of the same *Camellia* genus. Another representative example is that conducted by Wu et al. (2014) where leaf transcriptomes of the four tea plant cultivars, ‘Yunnan Shilixiang,’ ‘Chawan Sanhao,’ ‘Rucheng Maoyecha,’ and ‘Anji Baicha,’ with different percentages of various catechins, were subjected to a deep comparative analysis. In this effort, three catechin closely-related enzyme genes, ANS, ANR and LAR, were unraveled to be as key factors involved in the changed catechin percentages in different tea plant cultivars.

### Integrated Transcriptome and Metabolome Analysis

Single metabolome analysis applied in the tea plant is mainly focused on the accumulation patterns of chemical components in certain tea products [e.g., green (Lee et al., 2011) and black (Pan et al., 2017)] or plant parts [mainly leave (Pauli et al., 2016; Ryu et al., 2017)]. In these studies, the elucidation of metabolite-related genes has not been concerned. It is promising that the connection between gene expression and metabolite accumulation should be considered to study the ‘cause-to-effect’ relationships in the biosynthesis of specialized metabolites in plants because a metabolome in cell system represents the phenotype effect of gene actions. In the past few years, there has appeared several attempts that focused on integrated transcriptomics and metabolomics analysis in the tea plant, which is a popular fashion used from the model plant *Arabidopsis thaliana* to vegetables (Alba et al., 2005), fruits (Savoi et al., 2016), crops (Kovinich et al., 2011), and trees (Hamanishi et al., 2015). In a recent effort, Li et al. (2016) presented a combined transcriptomics and metabolomics analysis of ‘Anji Baicha’ (*Camellia sinensis*) leaves at yellow–green, albescent, and re-greening stages. In theanine biosynthetic pathway, one of the three characteristic metabolic pathways, the authors found that the expressions of four genes, GOGAT, AIDA, GS, and TS, were significantly correlated with the concentrations of ethylamine (*GOGAT*), glutamine (*GOGAT, AIDA, GS*, and *TS*), and theanine (*AIDA*) in this pathway, which are likely the causes of the leaf metabolite variability among the three color and developmental stages.

## Limitations of the Current Gene Discovery Strategies of Characteristic Metabolites in the Tea Plant in Contrast With ‘Omics’-Based Network Strategies

The exploration of genes responsible for characteristic components biosynthesis is an important branch of tea biochemistry research. Previously, characteristic metabolite genes were mostly discovered through Sanger sequencing (Park et al., 2004; Singh et al., 2009). With the advent of NGS technology (specifically RNA-seq), the gene decoding has made great achievements in the determination of gene structures and expression profiles relying on its high throughout and coverage superiority (Ozsolak and Milos, 2011). However, current RNA-seq based ‘omics’ analysis has several limitations in metabolite gene discovery by comparison with ‘omics’-based network approaches: (1) genes are always identified from assembled unigenes through homology-based function annotation based on well-characterized gene references in data-rich model species. This commonly used strategy in non-model species may shield the possibility in discovery of novel enzyme genes and the underlying regulatory TFs related to certain characteristic metabolic pathways in the tea plant. As emphatically discussed in this review, genes in a specific specialized pathway are usually co-regulated at the transcriptional level. Thus, it is logical that the gene-to-gene associations (e.g., gene co-expression network) at a genome-wide scale should be established to predict novel genes of a characteristic metabolic pathway in the tea plant based on the ‘guilt-by-association’ principle, using well-characterized bait genes. (2) Although integrated transcriptome and metabolome analysis can help disclose genes that contribute to certain metabolite accumulation pattern in certain biological conditions in the tea plant (see illustrated example above), the associations between genes and metabolites has still not been quantitatively measured into a gene-to-metabolite network (can be seen in other species) based on a multi-sample statistical model. That is to say, integrated transcriptome and metabolome analysis currently applied in the tea plant is intrinsically parallel-isolated at the two levels of gene expression and metabolite accumulation. (3) Among the three major characteristic components in the tea plant, theanine has been found in some *Camellia* species and in a mushroom, *Xerocomus badius* (Casimir et al., 1960). Therefore, the metabolic pathway associated with theanine biosynthesis has no reference pathway from other model plants to identify metabolite genes using the homology-based knowledge translation. (4) *Camellia sinensis* (the tea plant) is evolutionarily far-distant from *Arabidopsis thaliana* and other secondary metabolism well-established model plants. Thus, cross-species gene knowledge translation (homology-based) may hinder the elaborate disclosure of the specific biosynthesis of characteristic components in the tea plant.

## Potential of Introducing ‘Omics’-Based Network Strategies in the Identification of Characteristic Metabolite Genes in the Tea Plant

As summarized in section “A survey of ‘omics’-based network strategies for the identification of specialized metabolite genes in plants,” several prerequisites are required for the ‘omics’-based network strategies applied in the tea plant, such as sufficient sample size and well-characterized bait gene set in a certain characteristic metabolic pathway. We searched NCBI SRA (Leinonen et al., 2010b), a representative NGS sequence database, using the keyword “*Camellia sinensis*,” and manually checked biological samples that documented relevant RNA-seq applications in the tea plant. As of January 2018, more than 200 biological samples in the tea plant in different biological conditions are publicly available. In addition, dozens of in-house RNA-seq examples concerned with different biological questions have accumulated in our own lab in recent years. Thus, the RNA-seq sample size in the tea plant is now sufficient to allow for the statistical computation of co-expression relationships of pairwise genes [see sample requirement for gene co-expression decision in review (Aoki et al., 2007)]. As to bait genes, researchers in our tea lab at Anhui Agricultural University and colleagues around the world have contributed considerable efforts in biological molecular experiments that well-characterize genes related to the three characteristic metabolic pathways in the tea plant, such as UGT (Cui et al., 2016), *F3*′*H* (Zhou et al., 2016), GCH (Jiang et al., 2013), TS (Okada et al., 2006), and F3′5′H (Wang et al., 2014). Most recently, the genome of tea plant has been sequenced and released in public (Xia et al., 2017). Using this resource as a reference, we can accurately track genes and compute their expressions in diverse sequenced samples. In addition, researchers can also use the reference to computationally predict several molecular functional associations, such as protein–protein physical interaction and proteins subcellular co-localization, to enhance single-network of gene co-expression into multi-network of gene co-function. With respect to the gene-metabolite associations, parallel experimental design and integrated analysis related to gene expression and metabolite accumulation in concerned biological conditions can be now readily achieved for the possible gene-to-metabolite network inference in the gene identification of characteristic secondary metabolism of the tea plant.

## Possible Issues Regarding ‘Omics’-Based Network Approaches

When using ‘omics’-based network approaches for the identification of specialized metabolite genes in plants, several pitfalls and limitations should be concerned. As surveyed above, gene association network models (e.g., gene co-expression and co-functional networks) utilize the “guilt-by-association” principle to prioritize candidate genes that might be involved in a particular secondary pathway. However, genes in the same pathway are not necessarily co-expressed or have no co-functional relationships. For example, genes in a certain secondary pathway with post-transcriptional regulation may have no significant correlations among them in a gene model, and therefore they can’t be recognized using the above “guilt-by-association” method. More information about the limitation of the “guilt-by-association” principle in gene association network analysis can be seen in a critical article reported by Gillis’ group (Gillis and Pavlidis, 2012). When researchers integrate a large number of heterogeneous transcriptomic and/or metabolomic ‘omics’ data from public databases or their own collections in a network modeling pipeline, it should be noted that several technical issues such as batch effects and missing values intrinsically exist and may lead to misguided conclusions (Scherer, 2009). Inspiringly, there have appeared several computational techniques such as generalized R^2^ statistic model (Leek et al., 2010) and missing value imputation algorithm (Liew et al., 2010) that are developed to deal with batch effects and missing values. Another notification for users is the downstream experimental validation of selected candidate genes. False positive genes are possibly subjected to time-consuming biochemical test. Among the candidate genes, relevant ones can be manually screened out via the KEGG/GO (Gene Ontology Consortium, 2014; Kanehisa et al., 2015) functional annotation or other function database annotation and/or extensive literature reviewing of interest genes.

## Conclusion and Future Prospects

Considerable research examples demonstrate that ‘omics’-based network approaches are powerful tools for the gene discovery in plant-specialized metabolism (Hirai et al., 2004; Mercke et al., 2004). However, different ‘omics’-based network strategies for the identification of specialized metabolite genes in plants are not summarized in a single review article, which conceal the possible connections and differences of such systems biology approaches. Moreover, the advantages of such ‘omics’-based network strategies have not been discussed in a single paper by comparison with the traditional ‘omics’ analysis in the identification of specialized metabolite genes in plants. As to the tea plant focused in this review, apart from the high complexity in its genome, characteristic metabolic pathways in this crop have their intrinsic features different from other plant species. For example, as an ammonium-tolerant and perennial plant species, the tea plant is different from other plants in nitrogen metabolism, which systematically governs the three characteristic metabolic pathways and makes them particular (Britto and Kronzucker, 2002). Therefore, network-fashion systems biology approaches are necessary to be introduced to facilitate the *de novo* identification of key genes involved in characteristic metabolic pathways in the tea plant. With these considerations, this review focuses on the agriculturally important crop tea plant (can also extend to others plant species) in which characteristic secondary metabolites are primary determinants of tea quality and taste and key characteristic metabolite genes are still not fully understood. We highlight different ‘omics’-based network approaches for the gene discovery in plant-specialized metabolism using successful examples that are employed in model and non-model plants. Limitations of the traditional ‘omics’ strategies in discovery of specialized metabolite genes are discussed by comparison with ‘omics’-based network approaches using the tea plant as an instance. Particularly, the potential of introducing these strategies in the relevant field of the tea plant are particularly demonstrated. We believe this will provide novel directions in the exploration of functional genes associated with characteristic components in the tea plant, which is a critical basis for applied genetic improvement and metabolic engineering.

As useful large-scale computational pipelines, ‘omics’-based network approaches can provide clues about the potential candidate genes involved in certain characteristic metabolic pathways in the tea plant. Hence, a main concern is that tea biochemists should perform wet lab experiments to validate the predicted gene functions. Due to the complicated genetic background, the transformation system in the tea plant has still not been established. Thus, reverse genetics approaches, such as gene knockout and over-expressing, are not feasible for the experimental confirmation of identified gene functions in the tea plant. In such case, we can consider the correlation analysis of gene expression and metabolite accumulation in multi-samples statistical model to achieve it, following the regular molecular experiments such as clone, protein recombination, and enzymatic activity. With the reference genome of the tea plant currently available, an active research field of metabolite-based genome-wide association study [or mGWAS (Luo, 2015)] should be focused, as it is a good way to identify genomic loci associated to key metabolic pathways by re-sequencing a collection of tea plant cultivars or recombination lines around the world and profiling their metabolomes of tissues and conditions of interest. The loci information from mGWAS analysis can be considered together with the gene information inferred from ‘omics’-based network analysis to provide subtle clues for a specialized component biosynthesis in the tea plant. To date, detailed information regarding the bait genes in the three characteristic metabolic pathways in the tea plant has been scattered in published studies. As such, several tasks should be carried out to gain a comprehensive list of such bait genes, using manual curation from publications (Zhang et al., 2013) or more effective literature-mining tools (Jensen et al., 2006). In addition, exhaustive experimental characterization of characteristic metabolites genes as a more comprehensive bait gene list should be an ongoing program for gaining an optimized prediction. In the past 5 years, we have seen massive accumulation of transcriptome and metabolome datasets from different labs worldwide, which are valuable resources in tea secondary metabolism community. Thus, it is an urgent and promising task to develop the corresponding deposit and analysis platforms, which can aid in the network identification of characteristic component genes in the tea plant for experimental investigator without any bioinformatics skills. On this base, novel algorithms in biological network analysis, such as motif and module mining, should be borrowed to help find gene- and/or metabolite-mediated regulatory sub-structures [e.g., frequently-appeared feed-forward loop in plants, FFL (Sakuraba et al., 2015)] that might control a specific specialized metabolite pathway in the tea plant. In addition to the three characteristic components in the tea plant, there are also several other specialized metabolites with important nutritional values and health benefits, such as saponins (Sur et al., 2001) and volatile terpenes (Yang et al., 2013). Now it still remains as a virgin field that should call for the analogous studies in gene discovery to advance a comprehensive understanding of secondary metabolic profile in the tea plant.

## Author Contributions

SZ and XW conceived the project and wrote the manuscript. LZ and YT wrote the contents regarding metabolomics and transcriptomics. XW and C-TH provided scientific criticisms and manuscript proofreading.

## Conflict of Interest Statement

The authors declare that the research was conducted in the absence of any commercial or financial relationships that could be construed as a potential conflict of interest.

## Abbreviations

ADC: arginine decarboxylase
AIDA: alanine decarboxylase
ALT: alanine aminotransferase
ANR: anthocyanidin reductase
ANS: anthocyanidin synthase
CHI: chalcone isomerase
DEG: differentially expressed gene
DFR: dihydroflavonol 4-reductase
EGCG: epigallocatechin-3-gallate
F3H: flavanone 3-hydroxylase
F3′H: flavonoid 3′-hydroxylase
F3′5′H: flavonoid 3′,5′-hydroxylase
GC: gas chromatography
GCH: galloylated catechins hydrolase
GLS: glutaminase
GOGAT: glutamate synthase
GS: glutamine synthetase
GSL: glucosinolate
GST: glutathione *S*-transferase
LAR: leucocyanidin reductase
LC: liquid chromatography
MS: mass spectrometry
NGS: next generation sequencing
NMR: nuclear magnetic resonance
PAL: phenylalanine ammonia-lyase
PCC: Pearson correlation coefficient
RNA-seq: deep mRNA sequencing
TF: transcription factor
TS: theanine synthetase
UGT: UDP-glycosyltransferase
